# Membrane-bound IL-2 improves the expansion, survival, and phenotype of CAR Tregs and confers resistance to calcineurin inhibitors

**DOI:** 10.3389/fimmu.2022.1005582

**Published:** 2022-12-23

**Authors:** Jakob Kremer, Pierre Henschel, Daniel Simon, Tobias Riet, Christine Falk, Matthias Hardtke-Wolenski, Heiner Wedemeyer, Fatih Noyan, Elmar Jaeckel

**Affiliations:** ^1^ Department of Gastroenterology, Hepatology & Endocrinology, Hannover Medical School, Hannover, Germany; ^2^ Department I of Internal Medicine, Tumor Genetics, University Hospital of Cologne and Center for Molecular Medicine, Cologne (CMMC), University of Cologne, Cologne, Germany; ^3^ Institute of Transplant Immunology, Hannover Medical School, Hannover, Germany; ^4^ Institute of Medical Microbiology, Essen University Hospital, University Duisburg-Essen, Essen, Germany; ^5^ Department of liver transplantation, Multi Organ Transplant Program, University Health Network, University of Toronto, Toronto, ON, Canada

**Keywords:** regulatory T cells (Treg), chimeric antigen receptor (CAR), transplantation, graft tolerance, CNI resistance

## Abstract

**Background:**

Regulatory T cells (Tregs) play an important role in the maintenance of immune homeostasis and the establishment of immune tolerance. Since Tregs do not secrete endogenous IL-2, they are especially dependent on external IL-2. IL-2 deficiency leads to lower Treg numbers, instability of the Treg phenotype and loss of immune regulation. After organ transplantation, patients are treated with calcineurin inhibitors (CNIs), which further limits available IL-2. Application of low-dose IL-2 expands Tregs but also activates NK and CD8+ T cells. It was recently shown that graft-specific Tregs recognizing mismatched MHC I molecules *via* a chimeric antigen receptor were far more potent than polyclonal Tregs in the regulation of immune responses after solid organ transplantation in a humanized mouse model.

**Methods:**

Therefore, our aim was to enhance the function and stability of transferred CAR-Tregs *via* expression of membrane-associated IL-2 (mbIL-2).

**Results:**

mbIL-2 promoted higher survival, phenotypic stability, and function among CAR-Tregs than observed in clinical trials. The cells were also more stable under inflammatory conditions. In a preclinical humanized mouse model, we demonstrated that mbIL-2 CAR Tregs survive better in the Treg niche than control CAR Tregs and are even resistant to CNI therapy without affecting other Tregs, thus acting mainly in *cis*.

**Discussion:**

The functional and phenotypic improvements observed after membrane-attached IL-2 expression in CAR-Tregs will be important step for enhancing CAR-Treg therapies currently being tested in clinical trials for use after kidney and liver transplantation as well as in autoimmune diseases.

## Introduction

Regulatory T cells (Tregs) play a pivotal role in the maintenance of immune homeostasis and tolerance. While polyspecific Tregs were successfully used under lymphopenic conditions after stem cell transplantation, they did not show efficacy after adoptive transfer in the context of autoimmunity (type 1 diabetes; T1D) or after transplantation (The ONE Study). In contrast, graft- or tissue-specific Tregs have been successfully used in autoimmune models of T1D (1) and multiple sclerosis as well as after organ transplantation (2, 3). Due to the limited number of antigen-specific Tregs, these cells need to be massively expanded to obtain clinically relevant cell numbers. Furthermore, their use is limited by MHC restriction. Both the cell number challenge and MHC limitation can be overcome by genetically modifying natural Tregs (nTregs) with chimeric antigen receptors (CARs). With their target-specific antigen binding and signalling domains, these cells can restore the balance between effector T cells (Teffs) and Tregs at the site of action and induce tolerance after transplantation (2–4). The first clinical trial on the efficacy of CAR-Tregs after kidney transplantation was approved in the UK in 2019 (EudraCT No. 2019-001730-34, EU Clinical Trials Registry), and further trials focusing on liver transplantation are about to start (LIBERATE trial, NCT05234190).

An important factor in the number, function, and stability of Tregs is IL-2, which is not produced by Tregs themselves. Deficiency of IL-2 caused by defects in IL-2 signalling (5–7) or as a result of treatment with calcineurin inhibitors (CNIs) after transplantation leads to a loss of peripheral Tregs (8, 9). After adoptive transfer of Tregs in patients with T1D, their number decreased by up to 75% within 90 days (7, 10). This scarcity also represents a hurdle for adoptive Treg therapy, as no source of IL-2 is available for the transferred cells, and transfer further drastically lowers the existing serum IL-2 level (10).

In addition, Tregs may exhibit an unstable phenotype under proinflammatory conditions or when IL-2 is low. Instability of FOXP3 may lead to proinflammatory phenotypes of TH1- or Th17-like cells (11). Although low-dose IL-2 administration transiently increases Treg numbers and has been used with some success to treat autoimmune diseases, systemic administration enhances Tregs nonspecifically and may also activate CD8+ T cells and NK cells. Indeed, clinical studies in patients with T1D and in patients after liver transplantation have shown that systemic low-dose administration of IL-2 can lead to off-target adverse complications manifested by a decrease in C-peptide (7) or TH1 activation (EudraCT #2017-000177-37), resulting in graft rejection. This underscores the urgent need to develop immunotherapeutics that more effectively or exclusively target the Treg population. These include IL-2 muteins or complexed IL-2, which are more Treg specific. However, they still act on all Tregs nonspecifically. Therefore, our goal was to generate adoptively transferred graft-specific CAR Tregs with specific IL-2 signalling without transactivating Tregs of other specificities or proinflammatory cells.

Therefore, we developed a membrane-associated IL-2 (mbIL-2) that is anchored to the cell surface and allows autocrine activity. This approach was combined with successful antigen-specific targeting by graft-specific CARs.

## Materials and methods

### Human samples

Human specimens were obtained from different HLA-typed healthy donors. Local ethical committee approval was received for this study. Informed consent was obtained from all participating subjects.

### Human Treg cell isolation and expansion

Human natural Tregs (nTregs: CD4+CD25highCD127lowCD45RA+) were isolated from human peripheral blood mononuclear cells (PBMCs) after Ficoll (GE Healthcare) gradient separation and CD25 preenrichment (CD25 MicroBeads II, MiltenyiBiotech) *via* AutoMACS (MiltenyiBiotech). For fluorescence-activated cell scanning (FACS), PBMCs were labelled with monoclonal antibody combinations: CD4+ (RPA-T4, BioLegend), CD25+ (2A3, BD), CD127- (hIL-7R-M21, BD), and CD45RA+ (MEM-56, Thermo Fisher). HLA-A*02 status was confirmed by flow cytometry *via* α-HLA-A2/A28 (REA 142, Miltenyi Biotec). FACS-based cell sorting was performed at the cell-sorting facility of Hannover Medical School. The obtained purity of isolated Treg cells was >95%. Tregs were kept in TexMacs GMP cell culture medium (Miltenyi Biotech) supplemented with 10% human AB serum, 1% penicillin−streptomycin (Gibco), 1 mM sodium pyruvate, 1% nonessential amino acids (NEAA, Gibco), 20 mM HEPES and 50 µM beta-mercaptoethanol with the indicated concentrations of IL-2 (Proleukin, Clinigen). Tregs were expanded by using Treg expansion beads (MiltenyiBiotech) according to the manufacturer’s instructions.

### Cell transduction

For γ-retrovial transduction, isolated Tregs were first stimulated with plate-bound α-CD3 (5 µg/mL, UCHT1, BioLegend) and soluble α-CD28 (5 µg/mL, CD28.2, BioLegend) in complete medium for 48 hours. Before transduction, protamine sulfate (4 µg/ml, Sigma Aldrich) was added to the Treg cultures. Tregs were spin-infected with retroviral particles at 800 RCF and 37°C for 1 h. CD4+CD25highCD127lowCD45RA+ nTregs transduced with particles encoding the HLA-A*02 CAR and membrane-bound IL-2 were referred to as mbIL-2 CAR-Tregs, and those transduced with particles encoding solely HLA-A*02 CAR were referred to as CTR CAR-Tregs.

### Flow cytometry surface and intracellular staining

For determination of surface antigens, cells were washed and stained in PBS containing 0.5% w/v BSA and 2 mM EDTA plus monoclonal antibodies. For intracellular FOXP3 staining, the Foxp3 Transcription Factor Staining Buffer Set (eBioscience) was used according to the manufacturer’s instructions with α-FOXP3 (PCH101, eBioscience). pSTAT5 staining was performed by directly resuspending cultured cells for fixation in 1.5% formaldehyde for 10 min at RT followed by permeabilization in ice-cold methanol for 10 min at 4°C. Cells were stained with α-pSTAT5 (47/Stat5pY694, BD) as described above. Stained cells were analysed on an LSR II (BD) or CytoFLEX (Beckman Coulter) flow cytometer. Flow cytometry FCS files were analysed using FlowJo Software (version 10, BD).

### Cell lines

HEK293 cells were kept in DMEM (Gibco) supplemented with 10% FCS and 1% Pen/Strep. CTLL-2 cells were cultured in Treg medium (see above) supplemented with 50 U IL-2.

### CTLL-2 cell starvation assays

CTLL-2 cells were either transduced with the mbIL-2 CAR or left untransduced. Forty-eight hours post-transduction, the transduction efficiency was calculated based on ΔLNGFR expression. The biological effect of mbIL-2 on transduced CTLL-2 cells was assessed by direct comparison with CTLL-2 wild-type (WT) cells or in coculture assays (mbIL-2 CTLL-2 vs. CTLL-2 WT) under IL-2-limiting (0 U IL-2, starvation) or 100 U IL-2 conditions. Cells were counted 48 and 72 hours post-transduction.

### CAR-Treg cell starvation assay

Isolated Tregs were transduced with either the mbIL-2 CAR or CTR CAR and expanded as described above. On day 0, cells were washed, seeded and activated with the human Treg Expansion Kit (Miltenyi Biotec) according to the manufacturer’s instructions but with different amounts of IL-2: 1000, 25 or 0 U/mL. On days 7, 14 and 21, the cells were counted and stained for viability (Fixable Viability Dye, eBioscience), α-CD4 (RPA-T4, BioLegend; SK3, BD), a transduction marker (α-CD271/LNGFR, ME20.4, BioLegend; α-Human IgG, F(ab’)2, polyclonal, Jackson ImmunoResearch) and α-FOXP3 followed by flow cytometric analysis.

### CAR Treg cells under inflammatory conditions

Tregs were isolated and transduced with mbIL-2 CAR or CTR CAR as described above, followed by a resting period of 48 hours with 50 U IL-2/mL. Cells were stimulated with a human Treg Expansion Kit (Miltenyi Biotec) and cultivated in Treg medium supplemented with the following three cytokine mixes (11): control (10 U/mL IL-2 (Proleukin, Clinigen Inc.), Mix 1 (10 U/mL IL-2 (Proleukin, Clinigen Inc.), 10 ng/mL IL1β (BioLegend), 4 ng/mL IL-6 (BioLegend), 5 ng/mL TGF-ß (BioLegend)), and Mix 2 (10 U/mL IL-2, 25 ng/mL IL-21 (BioLegend), 25 ng/mL IL-23 (BioLegend), and 5 ng/mL TGF-ß). After 120 hours, the cells were harvested and stained for FOXP3 as described above.

### CAR Treg cells in *in vitro* proliferation assays under tacrolimus stimulation

Tregs were isolated and transduced with either mbIL-2 or CTR CAR or left untransduced, followed by expansion as described above and cultivation for 48 hours in 20 U IL-2/mL medium for 48 hours. Subsequently, the cells were stained with CFSE (Thermo Fisher Scientific). nTregs were left in Treg medium as a control, while transduced Tregs were stimulated with the human Treg Expansion Kit (Miltenyi Biotec) according to the manufacturer’s instructions plus either 5 ng/mL tacrolimus (Prograf, Astellas Pharma). After 192 hours, the cells were harvested, and cell proliferation was measured by flow cytometry.

### Animals

Nonobese diabetic (NOD)-RAG1^null^IL2c^null^ (NRG) mice aged 8 to 17 weeks old were used. All mice were bred and maintained under specific pathogen-free conditions at Hannover Medical School. All animal experiments were approved by the local Animal Ethics Review Board (Oldenburg, Germany, 11/0034, 16/2099, 21/3621, 16/2099, 21/3621) and performed in accordance with current German regulations.

### *In vivo* mbIL-2 CAR-Treg cell characterization in humanized mice

On day -10, NRG mice were reconstituted with 5×10^5^ HLA-A*02^-^ PBMCs. Reconstitution was monitored at 10 days post-reconstitution by staining for α-CD4 and α-CD8 (SK1, BioLegend) followed by flow cytometric analysis. On day 0, 3×10^5^ syngeneic CTR CAR Tregs and mbIL-2 CAR Tregs were injected at a ratio of 1:1. On day 1, 5×10 ^8^irradiated (60 Gy) HLA-A*02-positive PBMCs were transferred.

The effect of CNIs on mbIL-2 CAR-Tregs was assessed in reconstituted NRG-mice (see above). Additionally, 5×10^8^ HLA-A*02-positive PBMCs irradiated at 60 Gy were transferred on days 1, 3 and 5, accompanied by daily injection of tacrolimus (5 mg/kg). Spleens were removed on day 11, and cells were stained for human CD4 and CAR markers (α-IL-2, REA689, Miltenyi Biotec; α-CD34/RQR8, Qbend/10, Invitrogen) and analysed by flow cytometry. Controls lacking specific injections were performed as indicated in the experimental outline.

### CAR-Treg cell suppression assay

Tregs were isolated, CAR-transduced and expanded as described above. *In vitro* suppression assays were performed as previously described (2). CAR-Tregs were labelled with the cell proliferation dye eFluorTM 670 (Thermo Fisher Scientific). A total of 5×10^4^ syngeneic CD4+ and CD25- effector T cells were labelled with carboxyfluorescein diacetate succinimidyl ester (CFSE; 5 mmol/L), cocultured with mbIL-2 or CTR CAR-Tregs at various ratios and irradiated with 2×10^4^ allogeneic HLA-A*02-positive PBMCs for 5 days. Teff proliferation was calculated based on various Treg : Teff ratios *via* a CFSE dilution assay.

### Statistical analyses

Statistical analyses were performed using Prism Version 7 (GraphPad Software).

## Results

### Anchorage of IL-2 to the cell surface led to stable, membrane-associated expression of the cytokine

**Figure 1:**
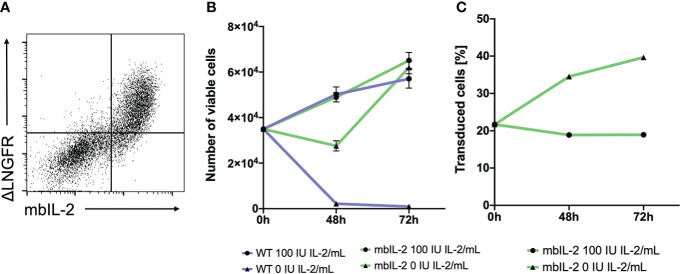
Anchorage of IL-2 to the cell surface led to stable, membrane-associated expression of the cytokine and mediated biological activity in CTLL-2 cells. **(A)** 293T cells were transfected with the mbIL-2 CAR vector plasmid to validate mbIL-2 surface expression. Representative dot plot confirmed coexpression of the ΔLNGFR CAR reporter gene and surface expression of mbIL-2. **(B)** The biological activity of mbIL-2 was assessed *via* the use of highly IL-2-dependent CTLL-2 cells. To address the possibility of autocrine IL-2 signalling, CTLL-2 cells were either transduced with mbIL-2 CAR or left untransduced (WT) and were cultured under IL-2-limiting conditions. Cultivation of CTLL-2 wild-type cells under IL-2-free conditions resulted in almost complete cell loss after 72 hours (9.2x10^2^ +/- 4.0x10^2^). The calculated number of mbIL-2 CTLL-2 cells (6.2x10^4^ +/- 2.7x10^3^) was comparable to that of CTLL-2 wild-type cells supplied with 100 U IL-2 (5.7x10^4^ +/- 4.1x10^3^). Culturing mbIL-2 CTLL-2 cells with 100 U exogenous IL-2 showed no negative effect on their cell growth (6.5x10^4^ +/- 3.5x10^3^). **(C)** The survival advantage of mbIL-2 CTLL-2 cells was compared to that of CTLL-2 WT cells in a competitive proliferation assay under IL-2-free conditions. At the starting point, mbIL-2 cells and CTLL-2 WT cells were cocultured at a ratio of 1:4. Under IL-2-free conditions, this ratio shifted towards mbIL-2 cells (1:1.5). Under physiological conditions for CTLL-2 cells with 100 U exogenous IL-2, no difference in cell growth between the two lines could be seen (n=3, error bars illustrate the mean +/- SD).

To fix the secretory cytokine IL-2 to the membrane, IL-2 was fused to the extracellular Gly_4_Ser linker, a transmembrane domain and the anchor domain of the MHC class I molecule. The length of the linker should allow activation in cis-, with no trans-activation (trans) of neighbouring cells (12). *Via* a 2A site, mbIL-2 was attached to the C-terminal end of the CAR signalling domain (**Supplementary Figure 1A**). Initial validation of mbIL-2 CAR surface expression was performed using 293HEK cells transfected with the retroviral mbIL-2 CAR vector. Flow cytometric analyses demonstrated coexpression of mbIL-2 and the reporter gene ΔLNGFR on the cell surface (**Figure 1A**). Using an HLA-A*02-PE dextramer as well as the ΔLNGFR-mAb, we successfully demonstrated that HLA-A*02-CAR expression correlated with IRES-directed ΔLNGFR expression. Based on our data, ΔLNGFR represented transduction efficiency as well as CAR expression (**Supplementary Figure 1B**).

### Membrane-bound IL-2 results in increased proliferation and survival of CTLL-2 cells

To validate the functionality of mbIL-2, CTLL-2 cells (13) were retrovirally modified with the mbIL-2 CAR vector. CTLL-2 is exclusively dependent on exogenous IL-2 for proliferation and survival (14). Transduced cells were cultured with different IL-2 concentrations (0 U or 100 U IL-2). In parallel, unmodified CTLL-2 wild-type cells were cultured under the same conditions to exclude the presence of active cytokines in the growth medium. While IL-2-free cultivation led to the death of all wild-type cells, the number of mbIL-2 CTLL-2 cells doubled during this period. Moreover, autocrine-guided mIL-2 signalling resulted in cell expansion under IL-2-limiting conditions that was comparable to that of CTLL-2 WT cells supplied with 100 U exogenous IL-2 (**Figure 1B**).

To directly compare the survival advantage of CTLL-2 cells with autocrine IL-2 signalling versus wild-type cells in an IL-2-free environment, these cell lines were cultured in a 1:4 competitive proliferation assay. The addition of exogenous IL-2 resulted in constant cell growth with no advantage to either cell line while maintaining the cell ratio originally used. This also demonstrated that there was no disadvantage of mbIL-2 cells in the presence of abundant external IL-2. In contrast, culturing cells in IL-2-free culture medium resulted in enhanced cell growth of mbIL-2 CTLL-2 cells. This resulted in a significant increase in the number of mbIL-2 CTLL-2 cells. The ratio shifted from 1:4 to 1:1.5 (**Figure 1C**).

### Expression of mbIL-2 in Tregs did not alter the Treg phenotype

**Figure 2:**
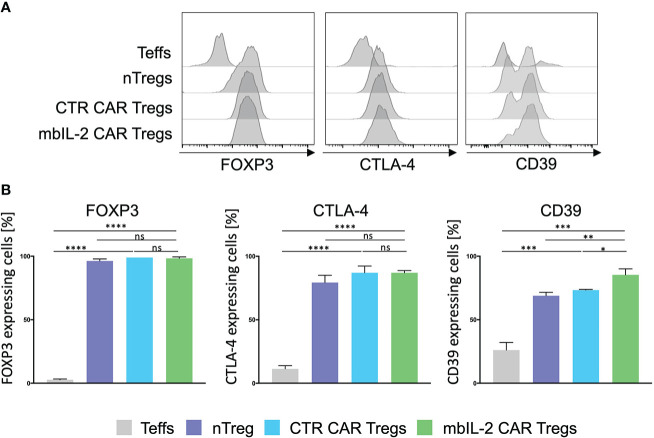
Expression of membrane-bound IL-2 did not alter the Treg phenotype. **(A)** Phenotype analysis of mbIL-2 CAR Tregs, CTR CAR Tregs and nTregs. CD4+CD25highCD127lowCD45RA+ Tregs from HLA-A*02–negative donors were transduced with mbIL-2 CAR Tregs or CTR CAR Tregs and compared with nTregs for the expression of FOXP3, CD39, and cytotoxic T-lymphocyte–associated protein 4 (CTLA-4). Representative histograms are shown. **(B)** Bars summarizing all three independent experiments. Data are shown as the mean ± SD (n=3, 3 different PBMC donors; unpaired, two-tailed Student’s t test, ns, not significant; *P ⪬ 0.0120, **P ⪬ 0.01, ***P ⪬ 0.0002, ****P ⪬ 0.0001).

The mbIL-2 CAR vector was used to transduce isolated CD4+CD25+CD127lowCD45RA+ nTregs from HLA-A*02-negative individuals (**Supplementary Figure 2**). mbIL-2 CAR-related modification did not change the nTreg phenotype. nTregs and mbIL-2 CAR Tregs showed similar levels of FOXP3 and similar levels of the effector molecule CTLA-4. CD39 was significantly increased in mbIL-2 CAR Tregs, suggesting that Tregs had a sustained FOXP3 expression level (15) (**Figure 2**). Furthermore, we did not observe any difference in the suppressive properties of mbIL-2 CAR Tregs compared to CTR CAR-Tregs in allogeneic suppression assays (**Supplementary Figure 3**).

### Membrane-bound IL-2 Tregs show better survival under IL-2-limiting conditions

**Figure 3:**
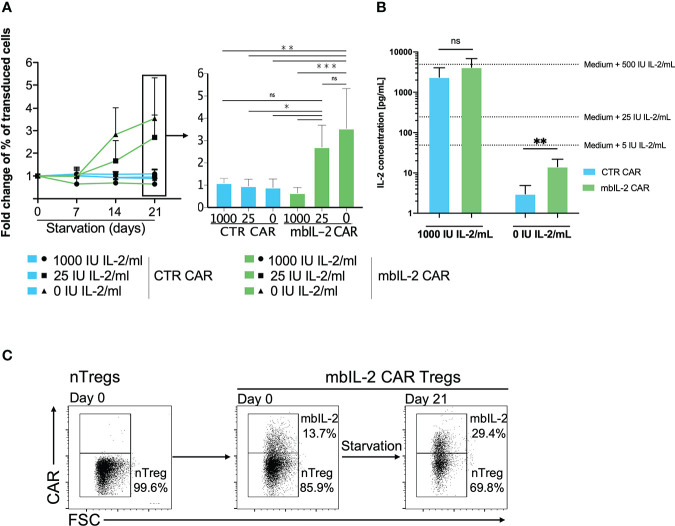
Membrane-bound IL-2 enabled Treg survival under IL-2-limiting conditions. **(A)** Left: Analysed fold change [%] of transduced cells for CTR CAR-Tregs and mbIL-2 CAR-Tregs at the indicated timepoints. Right: Bars represent a summary of all experiments performed on starvation day 21. mbIL-2 led to increased proliferation of mbIL-2 CAR-Tregs in IL-2-deprived or IL-2-free cultures. No difference in fold change was seen in mbIL-2 CAR-Tregs cultured with 25 U (2.70 +/- 0.99) or 0 U IL-2 (3.54 +/- 1.79) exogenous IL-2. In direct comparison, CTR CAR-Treg proliferation under nonphysiological conditions with 25 U (0.96 +/- 0.31) or 0 U (0.89 +/- 0.39) exogenous IL-2 was significantly lower (n=4, 3 different individuals). The normality of the distribution was assessed using the Shapiro−Wilk normality test. Significance was calculated with ordinary one-way ANOVA followed by Dunnett’s multiple comparison. Error bars show the mean ± SD. ns = not significant, *P ⪬ 0.05, **P ⪬ 0.005, ***P ⪬ 0.001. **(B)** Measurement of IL-2 concentrations in the supernatant of Tregs cultured in 0 U IL-2 medium using the BioPlex Cytokine 27-plex Assay system (Bio-Rad). Extrapolated medium IL-2 concentrations were calculated using control media with 1000 U IL-2/mL and 0 U IL-2/mL. Release of up to 14.33 pg/mL (+/- 7.54) was seen in mbIL-2 CAR-Treg culture medium. Significance was analysed using an unpaired t test. Error bars show the mean ± SD. ns = not significant, **P ⪬ 0.01. **(C)** To evaluate the effects of the soluble IL-2 concentration in the mbIL-2 CAR-Treg supernatant on nTregs, mbIL-2 CAR-Tregs and nTregs were cocultured at a ratio of 1:6 (14% vs. 86%) for 21 days under IL-2-free conditions. Flow analysis at day 21 revealed an increase in mbIL-2 of up to 30% and a concomitant shift in nTreg proportion of up to 70%, corresponding to a decrease in the original ratio to 1:2.3 (representative dot plots, n=3, 3 different individuals).

To investigate the biological effect of mbIL-2 expression in Tregs, the cells were maintained in expansion cocultures with nTregs under different conditions with different IL-2 concentrations (see **Figure 3**). The analysis was performed at different time points by comparing the cell number and the percentage of mbIL-2 CAR-Tregs.

While mbIL-2 CAR Tregs had no survival disadvantage under conditions of high exogenous IL-2 levels, their proportion increased significantly under limited IL-2 concentrations (25 U/ml, 0 U/ml). Because the cells were expanded for 21 days after transduction under high IL-2 levels, these effects were significant after 7 days under limited IL-2 concentration (**Figure 3A**). Moreover, mbIL-2 CAR Tregs continued to respond to high exogenous doses of IL-2, as expansion was higher under 1000 U/ml IL-2 than under 25 U/ml (data not shown) and retained their regulatory/suppressive capacity (**Supplementary Figure 3**).

Anchoring of initially secreted proteins to the cell surface may lead to their spontaneous release from the cell membrane by detachment or proteolytic cleavage, resulting in transactivation of neighbouring cells. Analyses of the supernatant showed that the initial IL-2-free cell culture medium of the mbIL-2 CAR-Tregs contained only 14 pg/ml soluble IL-2, even though the mbIL-2 CAR-Tregs were cultured for 21 days (**Figure 3B**). This concentration did not result in an *in vitro* expansion of nTregs and was consistent with the observed growth and survival of mbIL-2 CAR Tregs without apparent *trans* effects on nontransduced nTregs. Under IL-2-free conditions, nTregs tended to show a survival disadvantage despite the presence of cocultured mbIL-2 CAR Tregs, resulting in a decrease in Treg numbers. This precludes the possibility that mbIL-2 CAR Tregs were capable of autonomous growth and that IL-2 deposited from their cell surface had a beneficial effect on nTregs through cis-activation (**Figure 3C**).

### Expression of membrane-associated IL-2 led to STAT5 activation in the absence of exogenous IL-2

**Figure 4:**
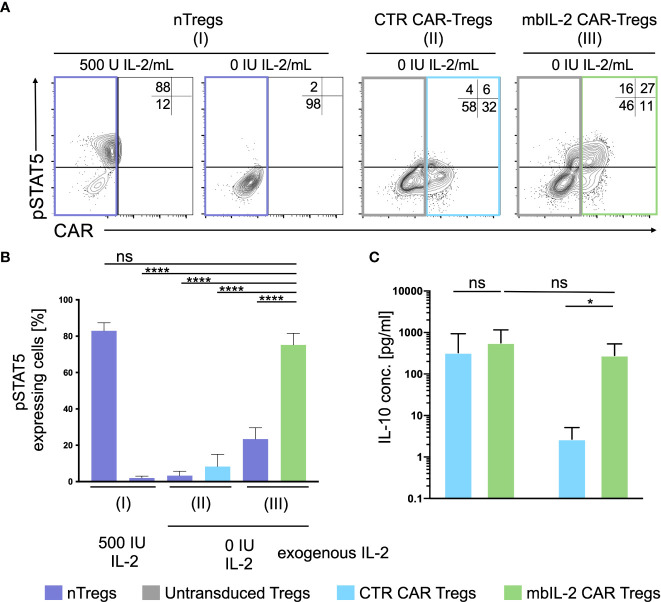
Expression of membrane-associated IL-2 led to STAT5 activation and enhanced IL-10 production in the absence of exogenous IL-2. **(A)** Representative dot plots of intracellular pSTAT5 staining in nTregs, CTR CAR-Tregs and mbIL-2 CAR-Tregs cultured under IL-2-free conditions. **(B)** Bar graph summarizes STAT5 activation correlated with IL-2-initiated signalling, which could be seen under IL-2-free conditions only in mbIL-2 CAR-Tregs (75,2 ± 2,8) and was comparable to that in nTregs cultured with 500 U IL-2 (83 ± 2,5). The absence of IL-2 signalling did not cause STAT5 activation, as seen in nTregs with 0 U IL-2 (2 ± 0,6) and CTR CAR-Tregs (8,3 ± 3,8). The percentage of pSTAT5 was calculated for either untransduced or transduced cells as indicated by the highlighted rectangles (n=3, 3 different individuals). Error bars show the mean ± SEM. Significance was analysed by ordinary one-way ANOVA with Dunnett’s multiple comparisons test. ****P ⪬ 0.0001, ns= not significant; each analysis was compared to pSTAT5 expression in mbIL-2 CAR Tregs. **(C)** Bar graph summarizes IL-10 measured concentrations in the supernatant of CTR CAR-Tregs and mbIL-2 CAR-Tregs cultivated under 500 U or 0 U exogenous IL-2. The absence of exogenously supplied IL-2 resulted in a decrease in IL-10 concentration. The presence of membrane-bound IL-2 in mbIL-2 CAR-Tregs stabilizes the IL-10 concentration even in the absence of IL-2 in the supernatant and is not significantly different from IL-10 concentrations measured in mbIL-2 CAR-Treg supernatant after culture with 500 U IL-2. The IL-10 concentration was measured using the BioPlex Cytokine 27-plex Assay system (Bio-Rad). n=3, 3 different individuals; significance was evaluated using an unpaired t test. Error bars show the mean ± SD. **P ⪬ 0.01, ns= not significant.

To define the potential of mbIL-2 CAR-Tregs in relation to activated STAT5, cells were characterized in the presence and absence of IL-2. Flow cytometric analyses showed an increase in pSTAT5 levels under exogenous IL-2 treatment that was not observed in the absence of IL-2. Whereas nTregs transduced with a control CAR did not show phosphorylation of STAT5, mbIL-2 CAR-Tregs showed strong STAT5 phosphorylation in the absence of exogenous IL-2 (**Figures 4A, B**). Up to 15-fold more pSTAT5 was detected in mbIL-2 CAR Tregs than in CTR CAR Tregs under IL-2-free conditions. More importantly, analysis of phosphorylated STAT5 in mbIL-2 CAR Tregs revealed comparably high expression of pSTAT5 in nTregs treated with 500 IU IL-2.

Membrane-associated IL-2 was thus able to establish STAT5 signalling in CAR-Treg cells independent of exogenous IL-2 and to provide pathways for stabilizing FOXP3 transcription in Tregs.

### IL-10 production by mbIL-2 CAR-Tregs is enhanced in the absence of exogenous IL-2

The release of inhibitory cytokines, cytolysis, and disruption of metabolism are just a few of the mechanisms by which Tregs maintain immunological balance (16). One of the suppressive mechanisms of Tregs is the release of immunomodulatory cytokines such as IL-10 (17), whose production is increased by STAT5 (18).

Under IL-2-free conditions, autocrine IL-2 signalling resulted in increased IL-10 production comparable to that in mbIL-2 cells treated with 500 U/ml IL-2 (**Figure 4C**). In contrast, secretion of the anti-inflammatory cytokine was not detected in CTR CAR-Tregs.

### No trans-activation of nTregs was observed in coculture experiments with mbIL-2 CAR Tregs

**Figure 5:**
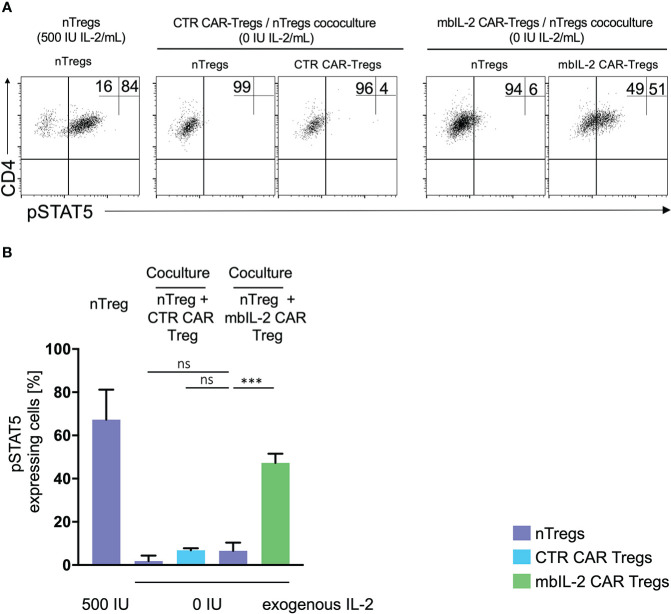
No transactivation of nTregs was observed in coculture experiments with mbIL-2 CAR-Tregs. **(A)** Representative dot plots of intracellular pSTAT5 staining in nTregs as well as in nTregs cocultivated with CTR CAR-Tregs and nTregs cocultivated with mbIL-2 CAR-Tregs under IL-2-free conditions. **(B)** Bar graph summarizing STAT5 phosphorylation in nTregs and various heterologous CAR/nTreg cocultures under IL-2-free conditions. pSTAT5 in nTregs (without cocultivation) was specifically upregulated in response to IL-2. 500 IU IL-2 resulted in pSTAT5 expression in 67.5% +/- 8.1 of cells, while under IL-2-free conditions, no pSTAT5 expression in Tregs could be detected (2%, data not shown). In coculture with CTR CAR-Tregs, pSTAT5 expression was not above the basal level under IL-2-free conditions in nTregs (1.8% +/- 1.4) or CTR CAR-Tregs (6.8% +/- 0.6). In contrast, phosphorylation of STAT5 in mbIL-2 CAR-Tregs was independent of exogenous IL-2, as shown. Under IL-2-free conditions, pSTAT5 was strongly upregulated in mbIL-2 CAR-Tregs (47.3% +/- 2.4). More importantly, nTregs cocultivated with mbIL-2 CAR-Tregs in the very same well did not benefit from mbIL-2-driven trans-activation, as only a weak pSTAT5 signal was seen in 6.6% +/- 2.2 of analysed nTregs (n=3, 3 different individuals, significance was calculated using an unpaired t test. Error bars show the mean ± SEM. Error bars show the mean +/- SEM. n=3, 3 different individuals; significance was analysed by ordinary one-way ANOVA with Dunnett’s multiple comparisons test. ***P=0.0002, ns= not significant.

In contrast to NK and CD8 cells, Tregs are equipped with a high affinity IL-2 receptor. This leads to sufficient STAT5 activation in environments with minimal IL-2 content. As shown previously, the expression of mbIL-2 in modified Tregs enables strong STAT5 phosphorylation in the absence of exogenous IL-2. To address the question of to what extent neighbouring unmodified Treg cells may benefit from this advantage by transactivating membrane-associated IL-2, heterologous mbIL-2 CAR-Treg/nTreg and CTR CAR-Treg/nTreg populations were characterized. For this purpose, modified and unmodified Treg cell populations were cocultured in the absence of exogenous IL-2. pSTAT5 was analysed by flow cytometry in each condition. STAT5 was phosphorylated in mbIL-2 CAR Tregs under such IL-2-free conditions, whereas a negligible pSTAT5 transactivation was seen in cocultured nTregs (**Figure 5**).

### Expression of membrane-bound IL-2 promoted a stable and high level of FOXP3 expression in CAR-Tregs

The stability of Tregs in organisms is one of the crucial factors for the maintenance of immune homeostasis (19).

**Figure 6:**
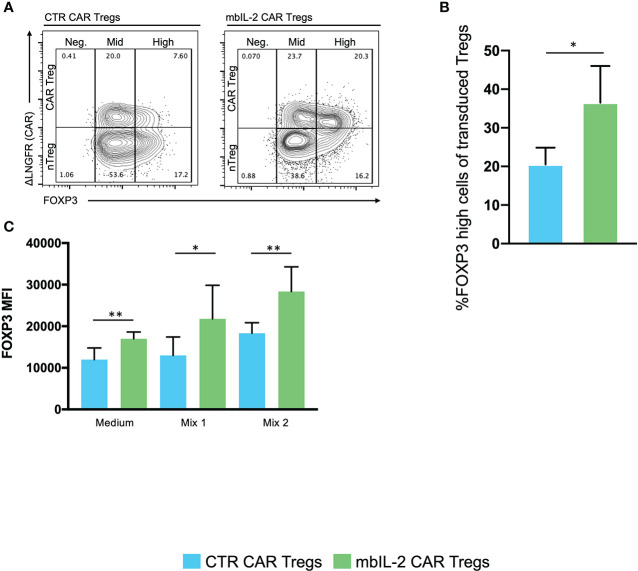
Expression of membrane-bound IL-2 resulted in high FOXP3 expression in mbIL-2 CAR-Tregs, protected them from conversion in the presence of the inflammatory cytokine mbIL-2 CAR-Tregs and protected them against the adverse effects of Tac *in vitro*. We analysed the levels of FOXP3 expression in either CTR CAR and nTregs or mbIL-2 CAR and nTregs in coculture assays. This mixed population was divided for the starvation experiment and further cultured under three different IL-2 concentrations (0, 25 U). On the indicated days, the cells were removed and analysed by flow cytometry. **(A)** Representative FACS plots illustrate the gating strategy for CTR CAR and mbIL-2 CAR-Tregs (0 U IL-2) in the starvation experiment with transduced and nontransduced Tregs and their classification into FOXP3-negative, -mid and -high cells. **(B)** Bar graph summarizes the percentage of FOXP3high cells within the Treg compartment of CTR CAR and mbIL-2 CAR-Tregs. By day 21, the proportion of FOXP3high cells in the mbIL-2 CAR-Treg compartment was nearly twice (36.50% +/- 9.54%) that in the CTR CAR-Treg compartment (20.50 +/- 4.36). n=4 of different individuals. Significance was evaluated using an unpaired t test. Error bars show the mean ± SD. *P ≤ 0.05. **(C)** Bar graph summarizes the mean fluorescence intensity (MFI) of FOXP3 expression in CTR CAR and mbIL-2 CAR-Tregs in the presence of cytokine mix 1 (IL-2, IL-1ß, IL-6, TGF-ß) and cytokine mix 2 (IL-2, IL-21, IL-23, TGF-ß). mbIL-2 CAR-Tregs showed a significantly higher FOXP3 MFI than CTR CAR-Tregs (1.3×10^4^ +/- 1.8×10^3^ vs. 2.2×10^4^+/- 3.2×10^3^, p value=0.0036). In combination with that observed for cytokine mix 2, a significantly higher MFI level was observed in mbIL-2 CAR-Tregs than CTR CAR-Tregs (1.8×10^4^+/- 1×10^3^ vs. 2.8×10^4^ +/- 2.4×10^3^). n=3, 3 different donors; significance was calculated using an unpaired t test. Error bars show the mean ± SEM. *P ⪬ 0.041, **P ⪬ 0.0035, ns = not significant.

Based on this, we assessed the impact of autocrine IL-2 signalling on FOXP3 expression/stability. Therefore, we analysed the levels of FOXP3 in either mbIL-2 CAR and nTregs or CTR CAR and nTregs in coculture assays under limiting IL-2 concentrations. The percentage of FOXP3high cells in the nTregs cultured with 25 U/ml IL-2 initially increased slightly until day 7 but then dropped to baseline levels. Under these conditions, the population of CTR CAR-Tregs cultivated with 25 U/ml IL-2 showed no change relative to a FOXP3high population. Similarly, in the absence of IL-2, only a minority of FOXP3-hi cells were observed in CTR CAR-Tregs and nTregs. In contrast, mbIL-2 CAR-Tregs with and without exogenous Il-2 had a high proportion of FOXP3-rich cells from day 7. By day 21, the proportion of FOXP3high cells was almost twice as high in the mbIL-2 CAR-Tregs (**Figures 6A, B**). Tregs can also lose Foxp3 expression and become unstable under inflammatory conditions (11). Therefore, we examined FOXP3 expression after exposure to inflammatory cytokines (20). mbIL-2 CAR-Tregs exhibited significantly higher FOXP3 expression in the presence of cytokine Mix 1, which became even more pronounced in the presence of cytokine Mix 2 (**Figure 6C**).

### mbIL-2 CAR-Tregs overcome the negative effects of tacrolimus *in vitro*

CNIs are part of the standard treatment for therapy after solid organ transplantation. Tacrolimus (Tac) impairs Tregs in a dose-dependent manner by directly inhibiting Treg activation (21).

**Figure 7:**
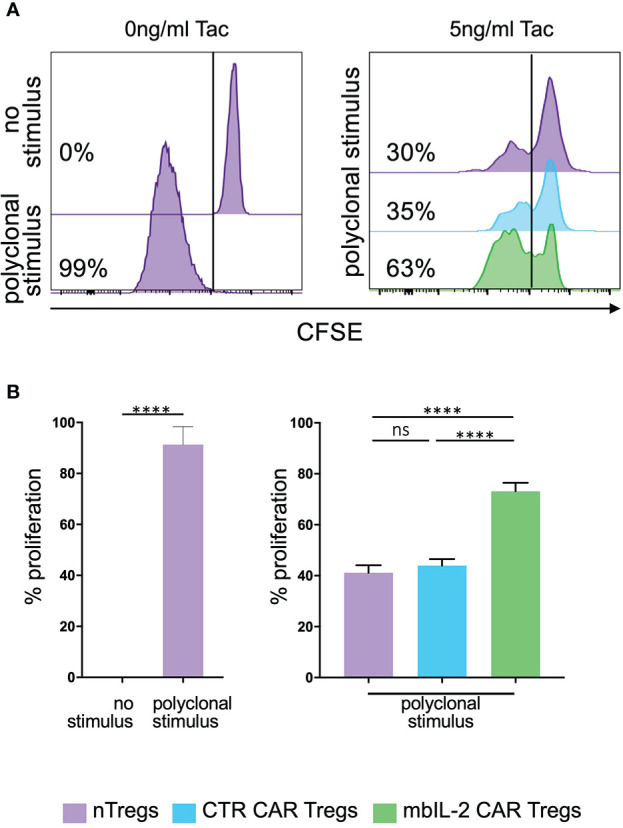
Expression of membrane-bound IL-2 protected against adverse effects of tacrolimus *in vitro*. **(A)** Representative histograms illustrating the proliferative capacity of polyclonally stimulated CTR CAR-Tregs and mbIL-2 CAR-Tregs in the presence of tacrolimus (Tac) and absence of exogenous IL-2. CTR CAR-Tregs and mbIL-2 CAR-Tregs were rested and labelled with a cell proliferation marker (CFSE). Left: representative histogram of unstimulated and polyclonal stimulated Tregs with no addition of Tac. Right: to assess proliferative capacity of mbIL-2 CAR Tregs after polyclonal stimulation in the presence of 5ng/ml tacrolimus, a CFSE dilution assay was performed. Cell proliferation was related to stimulated nTregs as well as to stimulated CTR CAR Tregs both under same conditions. **(B)** Bar graph summarizes all performed experiments. The proliferative capacity of mbIL-2 CAR-Tregs was significantly higher in the presence 5 ng/ml Tac (71% ± 4,9) in direct comparison to CTR CAR-Treg (44% ± 2,5; ^***^P value⪬0,0002) as well as to nTregs proliferation (41,2% ± 2,9; ^****^P value ⪬ 0,0001). No significant difference was found in the proliferation capacity of CTR CAR Tregs and nTregs (ns= not significant, P Value= 0,8448). Each analysis was compared to proliferation analysed in nTregs, cocultivated with mbIL-2 CAR-Tregs. Error bars show the mean ± SEM. Significance was calculated using ordinary one-way ANOVA with Tukey’s multiple comparisons test (n=3, 4 different donors).

To evaluate the effects of CNIs on Treg proliferation, CFSE-labelled mbIL-2 CAR- and CTR CAR-Tregs were stimulated in the presence of tacrolimus for five days with additional polyclonal stimuli. On day 5, CFSE dilution was used to determine cell proliferation (**Figure 7A**). CTR CAR-Treg proliferation was reduced in the presence of tacrolimus. In contrast, α-CD3/α-CD28-stimulated mbIL-2 CAR-Tregs showed much stronger proliferation, even in the presence of Tac levels equivalent to those observed in patients after transplantation. Notably, over 70% of mbIL-2 CAR-Tregs proliferated in the presence of 5 ng/ml Tac (**Figure 7B**). In direct comparison, mbIL-2 expression on CAR-Tregs was able to compensate for tacrolimus-induced proliferation inhibition.

### mbIL-2-enhanced CAR-Tregs showed enhanced survival *in vivo* after transplantation of allogeneic target cells in a preclinical humanized mouse model

**Figure 8:**
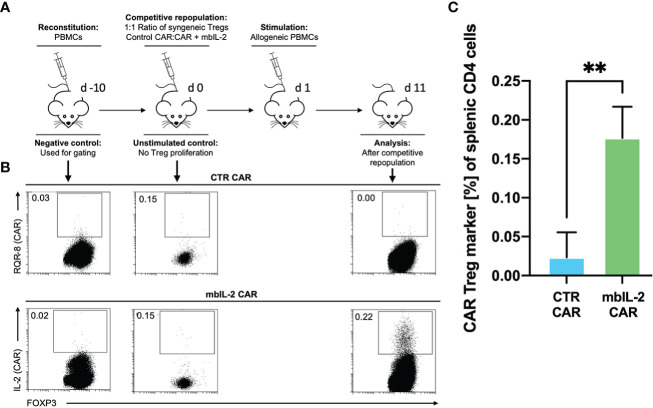
mbIL-2 enhanced CAR-Treg survival in an IL-2-limiting environment in a preclinical humanized mouse model. **(A)** Experimental setup. On Day -21, NRG mice were reconstituted with HLA-A*02negative PBMCs. Differently labelled syngeneic mbIL-2 CAR and CTR CAR-Tregs were adoptively transferred at day 0 in a 1:1 ratio followed by a single injection of allogeneic human HLA-A*02+ PBMCs to ensure a CAR-specific allogeneic stimulus. Spleens were isolated and analysed on day 10. **(B)** Representative dot plots show the decreasing fraction of CTR CAR-Tregs in the spleens of animals compared with that of mbIL-2 CAR-Tregs in this limiting IL-2 environment. **(C)** Bars summarize the data from all experiments. While a tenfold increase in mbIL-2 CAR-Tregs (0.18% +/-0.04%) could be observed, the number of CTR CAR-Tregs fell below the threshold value (0.02% +/- 0.03%). n=3 different mice. Significance was calculated using an unpaired t test. Error bars show the mean ± SD. P value ** ⪬ 0.01.

Having shown that autocrine IL-2 signalling exhibits increased proliferation and stability *in vitro*, we next evaluated its influence in a clinically relevant humanized mouse model. To this end, we evaluated the cell proliferation ability of mbIL-2 CAR and CTR CAR-Tregs in NRG mice in a competitive proliferation assay. Both CAR-Treg products were adoptively transferred at a 1:1 ratio into NRG mice stably reconstituted with syngeneic human PBMCs 14 days before the experiment (**Figure 8A**).

The next day, a single allogeneic CAR stimulus was initially performed with irradiated HLA-A*02-positive PBMCs. This ensured transient IL-2 production triggered by the allogeneic response. On day 11, the spleens were analysed for the presence of the applied Treg populations. While we observed a tenfold increase in mbIL-2 CAR-Treg numbers, CTR CAR-Tregs were no longer detectable (**Figures 8B, C**). This demonstrated the clear survival advantage of mbIL-2 CAR over CTR CAR-Tregs after stimulation by alloantigen *in vivo*. However, after a single antigenic stimulation, the majority of analysed Treg populations still belonged to the endogenous Treg repertoire. Uncontrolled proliferation of mbIL-2 CAR-Tregs could not be confirmed.

### mbIL-2 enabled CAR-Treg resistance to calcineurin inhibitors in a preclinical humanized mouse model

**Figure 9:**
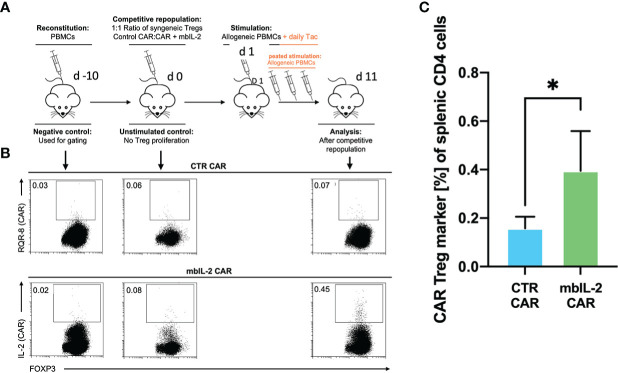
mbIL-2 enabled CAR-Treg resistance to calcineurin inhibitors in a preclinical humanized mouse model. **(A)** Experimental setup. On Day -21, NRG mice were reconstituted with HLA-A*02negative PBMCs. Differently labelled syngeneic mbIL-2 CAR and CTR CAR-Tregs were adoptively transferred at a 1:1 ratio. To ensure a continued allogeneic stimulus, animals received allogeneic HLA-A*02-positive PBMCs three times weekly. The scenario of maintenance therapy with CNI was mimicked by treating the animals daily with Tac (5 mg/kg). This resulted in an IL-2-poor/free environment. Spleens were isolated and analysed on day 10. **(B)** Representative dot plots show the decreasing fraction of CTR CAR-Tregs in the spleens of animals compared with that of mbIL-2 CAR-Tregs in this experimental scenario. The CAR-Treg gate was established according to the unstimulated negative control in animals killed one day after adoptive CAR transfer. It was found that 87% of all adoptively transferred cells could be assigned to the mbIL-2 CAR-Treg repertoire with autocrine IL-2 signalling and 13% to the CTR CAR-Treg repertoire. **(C)** Bars summarize the data from all experiments and illustrate the decreasing fraction of CTR CAR-Tregs compared with that of mbIL-2 CAR-Tregs. The proportion of CAR-Tregs that could be assigned to the mbIL-2 modification population (0.40% +/- 0.16%) was more than twice that of the CTR CAR population (0.16% +/-0.05%). n=4 of different experiments. Significance was calculated using an unpaired t test. Error bars show the mean ± SD. p* <0.05.

Whereas our initial *in vitro* experiments addressed the effect of Tac on Treg expansion, much of the *in vivo* effect of CNIs is mediated by blockade of IL-2 production by other T cells. Therefore, we sought to mimic the situation after organ transplantation by administering a repeated alloantigen stimulus in the presence of therapeutic levels of 5 mg/kg Tac. Animals were previously stably reconstituted with HLA-A2- PBMCs. Then, both mbIL-2 and CTR CAR-Tregs were adoptively transferred at a ratio of 1:1 in a competitive experiment to examine niche occupancy after adoptive transfer (**Figure 9A**). To ensure a continuous allogeneic immune stimulus, irradiated HLA-A2+ PBMCs were injected three times per week. On day 11, the spleens were analysed, and the original 1:1 ratio of CAR-Treg products was recalculated. It was found that 87% of all adoptively transferred cells could be assigned to the mbIL-2 CAR-Treg repertoire with autocrine IL-2 signalling and just 13% to the CTR CAR-Treg repertoire (**Figure 9B**). Indeed, CTR CAR-Tregs hardly expanded under the influence of Tac despite repeated allogeneic stimulation. In contrast, mbIL-2 CAR-Tregs expanded strongly despite the presence of Tac (**Figure 9C**).

## Discussion

Numerous studies have provided ample evidence for the potential of antigen-specific regulatory T cells in the treatment of autoimmune diseases and in transplantation medicine (22). Adoptive transfer of polyspecific Tregs showed clinical benefits only under lymphopenic conditions, such as those seen after haematopoietic stem cell transplantation. However, a therapeutic benefit in immunocompetent individuals has not been demonstrated thus far. In contrast, antigen-specific Tregs were proven safe in the context of autoimmunity (23, 24) and were much more potent in tolerance induction in models of autoimmunity (1, 25–27) and after transplantation (3, 28). Subsequently, redirection of Treg specificity by CARs has been shown to generate large numbers of Tregs independent of MHC restriction that can induce allotolerance even in the absence of immunosuppression (2–4). However, one of the remaining obstacles to their successful use is the obligatory persistence and maintenance of their regulatory phenotype in the context of autoimmunity (29–32). These scenarios leading to depletion of the Treg repertoire are caused by drastically reduced IL-2 levels or unstable FOXP3 expression (33).

Newer studies indicated that CsA in combination with low-dose IL-2 administration resulted in an increase in Tregs (34), which may indicate that the desired effect of selective Teff inhibition can be achieved while sparing Tregs. Although this statement is controversial discussed (35, 36), the use of tacrolimus currently represents the standard of care or CNI inhibitor of choice in patients after liver transplantation (37). After solid organ transplantation, CNIs reduce IL-2 levels within the graft, thereby counteracting Treg-mediated natural tolerance mechanisms (38).

Previous approaches to overcome this Treg gap by recombinant IL-2 substitution have failed thus far in clinical trials.

While low doses of IL-2 were initially successfully used in the context of autoimmune conditions such as alopecia and vasculitis, further trials revealed that there is a delicate balance between tolerance and immunity even with low-dose IL-2.

Indeed, patients with T1D treated with low-dose IL-2 showed declining c-peptide levels (7). In addition to an increase in peripheral Tregs, IL-2 also activated CD8+ T cells and NK cells. Likewise, a recent trial aiming at weaning from immunosuppression after liver transplantation under low-dose IL-2 (LITE trial clinicaltrials.gov) had to be stopped as patients developed T-cell-mediated rejection. In fact, higher peripheral Treg numbers do not always correspond to increased Treg numbers within the graft.

Other approaches for Treg-adapted IL-2 substitution acting more specifically on Tregs, such as IL-2 muteins (39) or antibody complexed IL-2 (31, 40), are being used to improve the specific binding and selectivity of Tregs (41). Because of the lower affinity or availability of IL-2R, these constructs preferentially act on CD25-expressing cells such as expanding Tregs. Indeed, it has been shown that these approaches can be used to treat autoimmune diseases (31) and that they enhance adoptive Treg therapy after transplantation (42). However, under inflammatory conditions, patients will also accumulate CD25-expressing Teffs and NKs, which could then be activated by these therapeutics. In addition, these second-generation IL-2 therapies enhance Tregs with various specificities.

We therefore provided our CAR Tregs with this essential Treg signal for survival by expressing a membrane-bound IL-2 that bypassed the IL-2 dependence of the antigen-specific Tregs. These mbIL-2 CAR Tregs resisted the destabilizing impact of proinflammatory cytokines or drugs for maintenance therapy at the site of action. Because we used an appropriate short, flexible linker, our membrane-bound IL-2 signal exclusively acts on the transferred mbIL-2 CAR-Tregs in the *cis* direction, providing the cells with a distinct survival advantage and a stable phenotype in the local Treg niche.

By using this technique, we were able to generate autocrine signalling in mbIL-2-Tregs without abandoning sensitivity to exogenous IL-2. In contrast to recent low-dose IL-2 therapies, the biochemical radius of mbIL-2 CAR- Tregs was strictly limited to the expressing cells as well as to the site of action due to their antigen specificity. Transactivation or accumulation of shed IL-2, which would have led to systemic activation, could be excluded, even after prolonged cultivation, in adjacent nonmodified cells. The spatial restriction of CAR-Treg activity is based on the directed specificity against the corresponding autoimmune or alloantigen and is exclusively linked to a CAR stimulus, preventing an uncontrollable systemic mode of action.

Although antigen-specific Tregs are much more potent (43–45), their therapeutic use carries the risk of an unstable phenotype, potentially giving rise to tissue-specific effector T cells (11, 46), preferably under either proinflammatory (11) or low IL-2 conditions (47).

IL-2 signalling is directly linked to the Treg phenotype *via* the IL-2/JAK3/STAT5 signalling pathway and ensures the initiation and maintenance of FOXP3 (48). IL-2R signalling is primarily mediated by the activation of JAK1 and JAK3, with the subsequent phosphorylation and activation of STAT3 and STAT5 (49). The major role in maintaining FOXP3 expression is attributed to STAT5, which in turn leads to its maintenance *via* complex signalling with IL-2 (50). STAT5 binds to specific binding sites around the FOXP3 enhancer region CNS2 and enables its transcription (51, 52). While proinflammatory conditions antagonize FOXP3 expression in inflamed areas, mbIL-2 was able to counteract this effect by stabilizing IL-2/STAT5 signalling, leading to interaction with CNS2 and restoration of stable FOXP3 expression. This allows the maintenance of the regulatory phenotype of CAR-Tregs in hotspots of inflammation and counteracts the conversion of Tregs to Teffs and prevents the potentiation of the effector immune response (53–56). In fact, the FOXP3 expression of these cells was much higher than that of neighbouring Tregs, and they produced more IL-10.

It has been shown that the transfer of large numbers of Tregs does not necessarily improve their *in vitro* survival (29), possibly due to the limited amount of IL-2 after transfer. Thus, the transferred mbIL-2 CAR-Tregs would have an intrinsic survival advantage within the endogenous Treg niche to avoid expansion under GMP conditions. This was shown in experiments investigating competitive survival under IL-2-free conditions in humanized mice. mbIL-2 CAR-Tregs and CTR CAR-Tregs were administered at a 1:1 ratio and stimulated once with alloantigens. Under these IL-2-limited conditions, only mbIL-2 CAR-Tregs expanded in the Treg niche, whereas very few CTR CAR-Tregs survived.

What are the consequences for adoptive Treg therapies after organ transplantation, such as the ONE study or ongoing trials with CAR Tregs after kidney or liver transplantation (57)? In all these trials, CNIs form the backbone of immunosuppression after transplantation. Accordingly, it is not advisable to omit CNI administration in this scenario. However, this will lead to the formation of a Treg-hostile environment. Calcineurin inhibitor administration deprives the regulatory arm of the immune system of cardinal factors and supports organ preservation by simultaneously suppressing Teff immune activation. This is precisely the pitfall of CAR-Treg therapy in organ transplantation. It is therefore of major importance that mbIL-2 CAR-Tregs show resistance to CNIs under physiological levels of tacrolimus mimicking trough levels in patients. The expression of mb-IL-2 minimizes the effect of CNIs on Tregs but more importantly creates a niche for mbIL-2 CAR-Tregs *in vivo*, even with the daily use of tacrolimus, by promoting less dependence on Teff-derived IL-2. Indeed, we tried to mimic the effect seen after organ transplantation by repeated transfer of allogeneic PBMCs in our humanized mouse model and daily application of Tac. The number of CTR CAR-Tregs was barely maintained in the niche under these conditions in cotransfer experiments, demonstrating the clear *in vivo* survival advantage of mbIL-2 CAR-Tregs. Resistance to CNIs makes mbIL-2 CAR-Tregs preferred candidates for experiments with Tregs after solid organ transplantation, supporting the interruption of immunosuppression.

IL-2 remains a double-edged sword in the balance between tolerance and immune activation, and prediction of the immunological balance and associated therapeutic effect remains challenging. Low-dose IL-2 therapies have thus far failed to provide benefit to nTregs without activating the immune system. Impressively, it was shown here that membrane-associated IL-2 could dramatically improve the stability of CAR-Tregs. Thus, for the first time, CAR-Tregs had a spatial advantage and no longer relied on exogenous IL-2. Because of the resulting CNI resistance, this provides new opportunities for the future use of antigen-specific Tregs for tolerance induction after transplantation and in the context of autoimmunity.

## Data availability statement

The datasets presented in this article are not readily available because Pending Patent Application. Requests to access the datasets should be directed to noyan.fatih@mh-hannover.de.

## Ethics statement

Local ethical committee approval was received for the studies. Informed consent of all participating subjects was obtained. The patients/participants provided their written informed consent to participate in this study. The animal study was reviewed and approved by Local Ethics Animal Review Board (Oldenburg, Germany, 11/0034, 16/2099, 21/3621, 16/2099, 21/3621).

## Author contributions

Study concept and design (FN, EJ); acquisition of data (JK); analysis and interpretation of data (JK, FN, EJ); drafting of the article (FN, EJ); critical revision of the article for important intellectual content (JK, PH, DS, MH-W, TR, CF, HW, FN, EJ); statistical analysis (JK); acquisition of funding (FN, EJ); administrative, technical, or material support (PH, DS, MH-W, TR, CF, HW); and study supervision (FN, EJ). All authors have read and agreed to the published version of the manuscript.
