# Supplementary figures and images for: Membrane-bound IL-2 improves the expansion, survival, and phenotype of CAR Tregs and confers resistance to calcineurin inhibitors
Source: Front Immunol. 2022 Dec 23;13:1005582. doi: 10.3389/fimmu.2022.1005582 (PMC9816406; doi:10.3389/fimmu.2022.1005582)

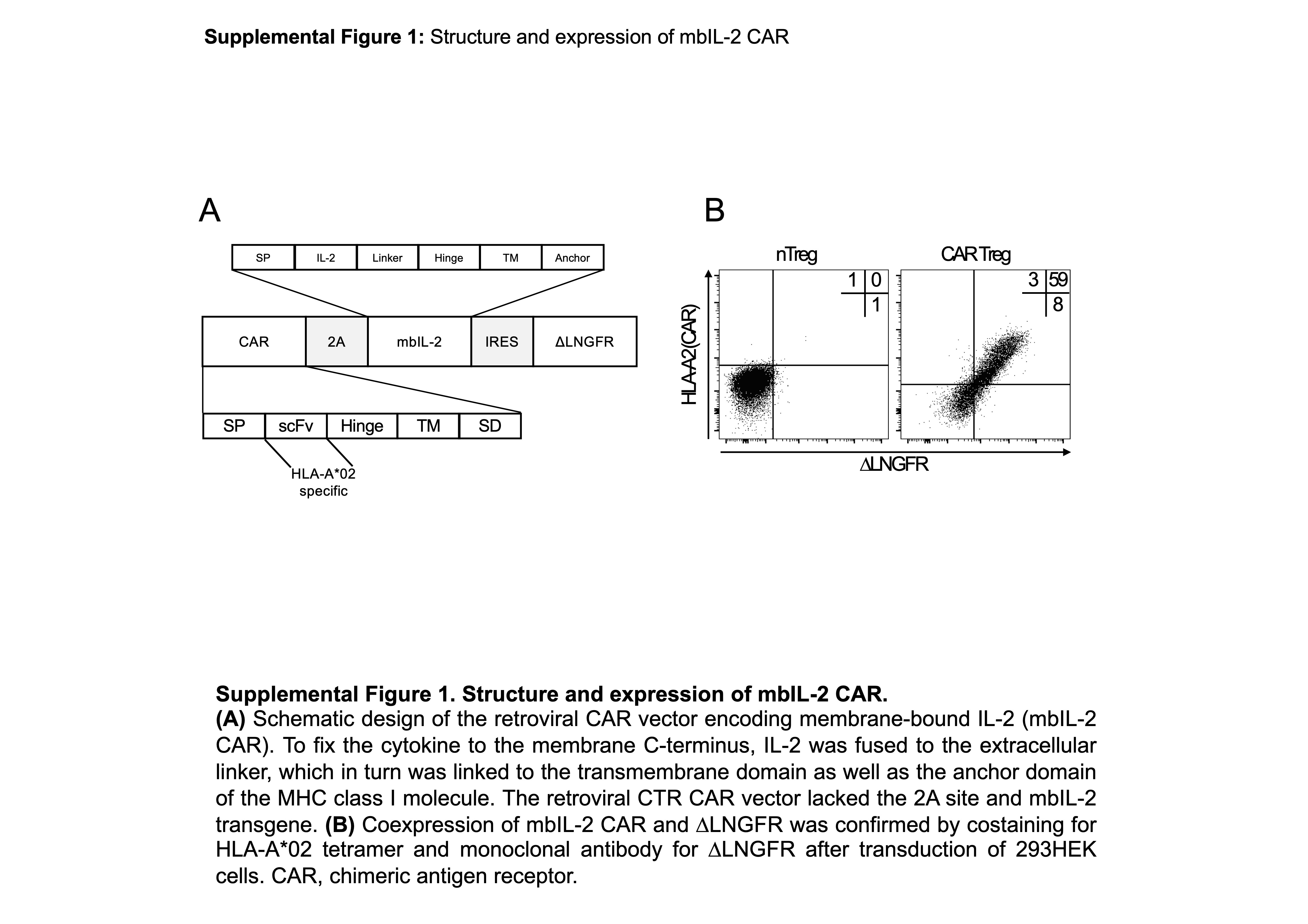

Supplement: Supplementary file 1 [file Image_1.jpg]

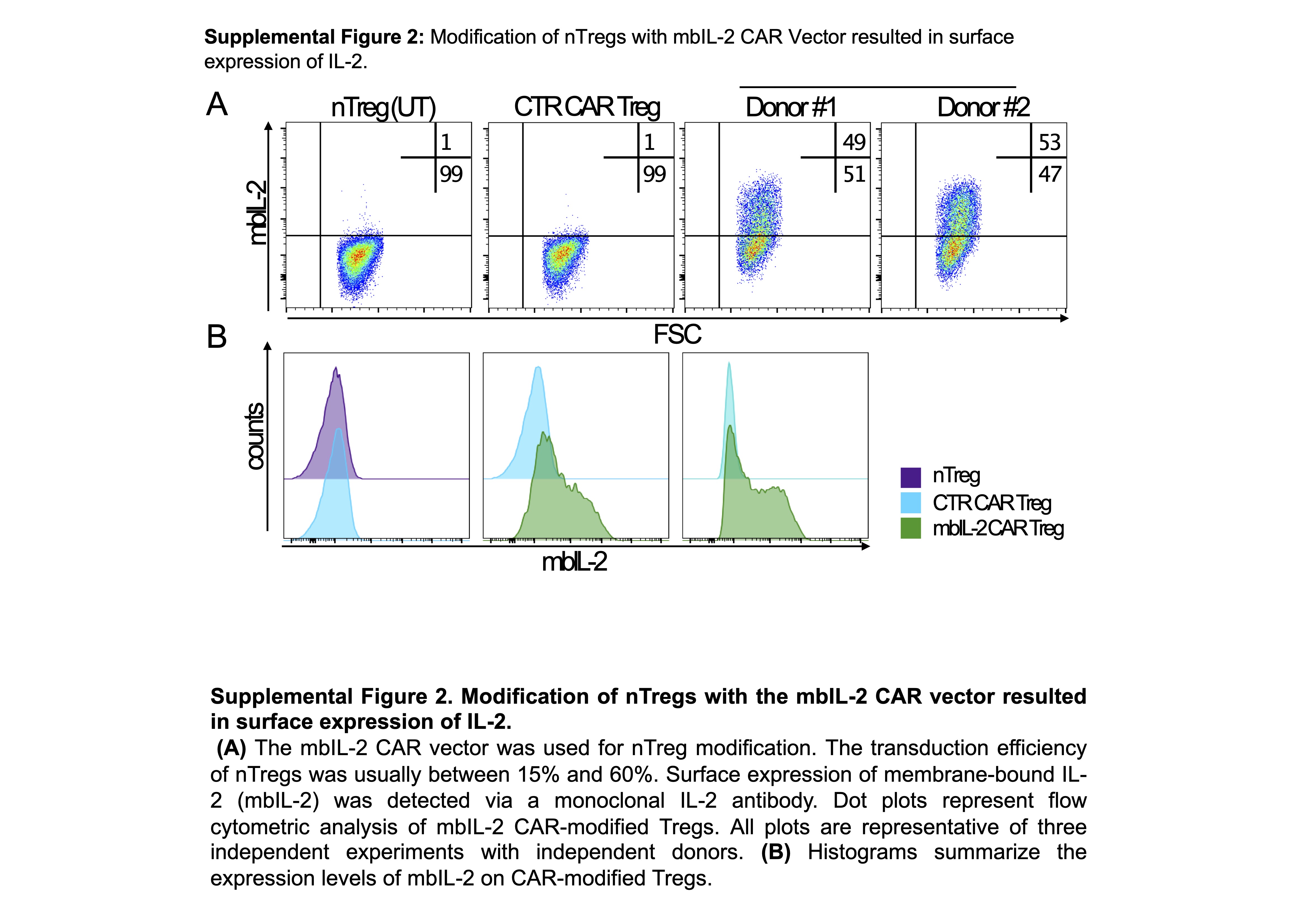

Supplement: Supplementary file 2 [file Image_2.jpg]

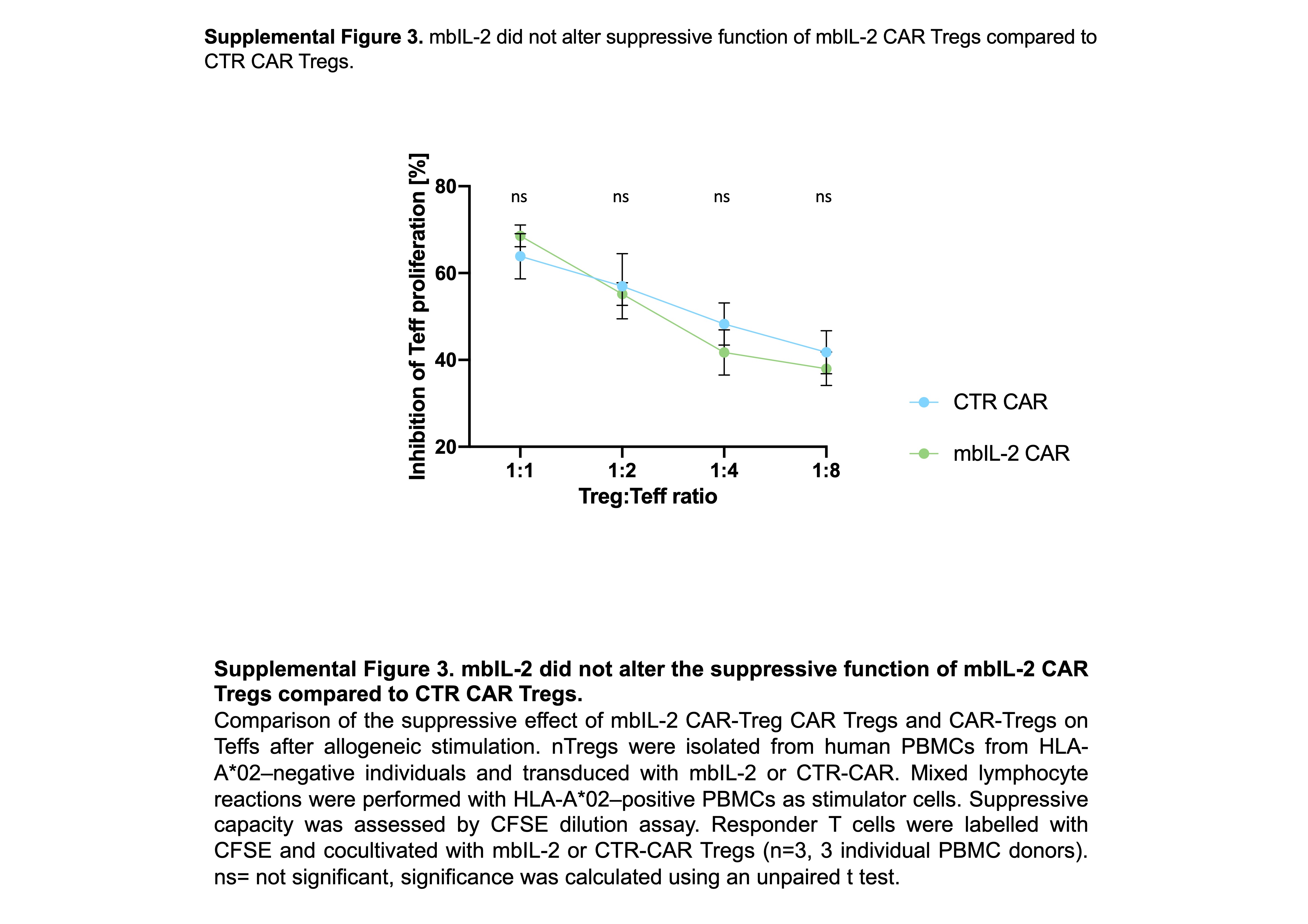

Supplement: Supplementary file 3 [file Image_3.jpg]
